# Cellular and Molecular Regulation of Spiral Artery Remodelling: Lessons from the Cardiovascular Field

**DOI:** 10.1016/j.placenta.2010.03.002

**Published:** 2010-06

**Authors:** G.St.J. Whitley, J.E. Cartwright

**Affiliations:** Developmental and Endocrine Signalling Centre, Division of Basic Medical Sciences, St. George's, University of London, Cranmer Terrace, London, SW17 0RE, UK

**Keywords:** Spiral artery, Trophoblast, Remodelling, Endothelial, vascular smooth muscle, Apoptosis, Extracellular matrix, pregnancy

## Abstract

A number of important changes take place in the maternal uterine vasculature during the first few weeks of pregnancy resulting in increased blood flow to the intervillous space. Vascular endothelial and smooth muscle cells are lost from the spiral arteries and are replaced by fetal trophoblast cells. Failure of the vessels to remodel sufficiently is a common feature of pregnancy pathologies such as early pregnancy loss, intrauterine growth restriction and pre-eclampsia. There is evidence to suggest that some vascular changes occur prior to trophoblast invasion, however, in the absence of trophoblasts remodelling of the spiral arteries is reduced. Until recently our knowledge of these events has been obtained from immunohistochemical studies which, although extremely useful, can give little insight into the mechanisms involved. With the development of more complex in vitro models a picture of events at a cellular and molecular level is beginning to emerge, although some caution is required in extrapolating to the in vivo situation. Trophoblasts synthesise and release a plethora of cytokines and growth factors including members of the tumour necrosis factor family. Studies suggest that these factors may be important in regulating the remodelling process by inducing both endothelial and vascular smooth muscle cell apoptosis. In addition, it is evident from studies in other vascular beds that the structure of the vessel is influenced by factors such as flow, changes in the composition of the extracellular matrix, the phenotype of the vascular cells and the local immune cell environment. It is the aim of this review to present our current knowledge of the mechanisms involved in spiral artery remodelling and explore other possible pathways and cellular interactions that may be involved, informed by studies in the cardiovascular field.

## 1 Introduction

In human pregnancy fetal trophoblast cells invade into the wall of the uterus in a tightly regulated manner. Cytotrophoblast cell columns form from the tips of anchoring villi and a shell develops from which extravillous trophoblasts invade into the decidua. Interstitial extravillous invasion occurs through the decidua with interstitial cells reaching the superficial myometrium by the eighth week of gestation [1]. Endovascular extravillous invasion arises as cells from the cytotrophoblastic shell invade the uterine spiral arteries from sites where they lie over the distal openings of the vessels, with endovascular cells detectable in decidual segments of the spiral arteries from approximately 8 weeks but especially after 10 weeks [2]. Invasion by extravillous trophoblast causes a temporary plugging of spiral arteries, decreasing flow of maternal blood which may protect the fetus from oxidative stress [3]. Dissolution of these plugs may trigger further trophoblast invasion [4] and, from 14 weeks, endovascular trophoblast can be seen in deeper spiral artery segments of the inner myometrium [5].

The blood supply to the uterus comprises a branched structure with successive decreases in vessel diameter as they progress through the myometrium and endometrium. Spiral arteries, so called because of their coiled appearance, supply blood to the endometrial layer and, in the pregnant uterus, span the inner myometrium and the decidua. During pregnancy the placental bed spiral arteries are transformed from high-resistance, low-flow vessels into large dilated vessels with an increased blood flow at a much reduced pressure [6]. These alterations occur as a result of loss of smooth muscle cell and elastic lamina from the vessel wall. The endothelium is temporarily replaced with a trophoblast layer, although it is restored later in pregnancy. The lumen of the remodelled vessel is dilated and there is trophoblast deposition of a fibrinoid matrix in the vessel wall; together these alterations are termed “physiological change” [7].

In resistance vessels such as the spiral artery, vascular smooth muscle cells (VSMC) are coiled round a central lumen formed by a single layer of endothelial cells (EC). At the distal end of spiral arterioles the wall is formed from a single layer of VSMC while in more proximal segments this can be several cells thick. Myometrial spiral arteries have a luminal diameter of approximately 200 μm [8] while remodelled vessels at the point of discharge into the intervillous space can attain a diameter of 2 mm [9]. These dramatic changes to the vessel architecture result in a tenfold increase in the blood supply to the fetoplacental unit in the third trimester compared to the non-pregnant uterus [10]. It is thought that virtually all 100–150 arteries in the placental bed are transformed [11] with spiral arteries at the centre of the placenta being more extensively remodelled than those at the periphery [7]. How our knowledge of the changes that occur at the fetal–maternal interface has developed together with some of the controversies in the field have been extensively reviewed elsewhere [4,12].

## 2 Trophoblast invasion and spiral artery remodelling

Extravillous trophoblast invasion can either be interstitial through the decidua or endovascular via the distal ends of the spiral arteries where invasion occurs retrograde to blood flow. In the study of spiral artery remodelling immunohistochemical studies have been informative; however, a number of questions remain unanswered regarding the relative importance played by the different routes of trophoblast invasion [4]. Decidua-associated vascular remodelling, including endothelial vacuolation and VSMC swelling, occurs prior to trophoblast invasion and may be under the control of the same factors responsible for initial changes in the decidua, such as steroid hormones. Recent immunohistochemical studies indicate that immune cells may play a role at this stage [13]. These changes are followed by interstitial trophoblast invasion which, it is suggested, influences the cells of the vessel wall by preparing them for subsequent endovascular invasion [5]. This concept is supported by studies of decidual vessels [14] where it was shown that medial disruption only occurred when interstitial trophoblast were present around the vessel. Conceptually, an initial interaction of interstitial trophoblast with VSMC is logical given the location of the cells, and is supported by observations that smooth muscle cells were lost from the external layers of the vessel media first [14]. In this context, the effects on medial organisation and extracellular matrix proteins may be particularly important. Trophoblast invasion occurring via the endovascular route would facilitate initial interactions with the endothelium. Indeed, trophoblast and endothelial cells have been shown to transiently co-exist in vessels undergoing remodelling [15]. Following the loss of the endothelium these endovascular trophoblasts would then be positioned to further influence medial VSMC.

It has been a matter of some debate as to whether the endovascular trophoblast has a route of invasion that is separate and distinct from the interstitial trophoblast. It has been suggested that the endovascular trophoblast arises from the interstitial trophoblast invading through the vessel wall, rather than migration intraluminally [16]. This theory has been refined to suggest that, although this may be the case in the more superficial decidua, it is unlikely to account for the presence of endovascular cells further into the myometrium where there are many fewer perivascular interstitial cells [4]. A combination of both routes may, therefore, be important, with their relative input determined by where the invasion is taking place spatially within the placental bed.

Regardless of the route, it has been well established that trophoblast invasion can be influenced by a plethora of regulating factors such as cytokines and growth factors, adhesion molecules, matrix metalloproteinases (MMPs) and oxygen tension (reviewed by Lunghi et al. [17]). In addition, the local cellular environment will influence this (for example, maternal immune cells present in the decidua such as macrophages and decidual natural killer cells) either through direct interactions or by secretion of soluble factors. These factors not only influence the invasive process but also influence the phenotype of the trophoblast, for instance immunohistochemical studies suggest that there are changes in trophoblast expression of various adhesion molecules as they near spiral arteries, perhaps in preparation for their interaction with and replacement of the vascular cells [18]. The orchestrated movement of trophoblasts is illustrated by in vitro co-culture studies which demonstrate that trophoblast migration towards vascular cells is directional [19] and that trophoblasts preferentially target arteries rather than veins [20].

## 3 Impaired remodelling in pre-eclampsia

In pregnancies complicated by the common pregnancy disorder pre-eclampsia, remodelling is restricted to the decidual regions of the spiral arteries [21]. The altered uteroplacental haemodynamics, as a consequence of impaired vessel remodelling, has been implicated as a contributing factor to the pathology of this syndrome. Similar deficiencies have also been described in spiral arteries of women with small-for-gestational-age infants [22].

Impaired invasion is likely to be a component of pre-eclampsia but with potential differences in the interstitial versus endovascular cells. Interstitial invasion did not appear impaired in initial studies [23], suggesting that defects may exist in the endovascular trophoblast invasion pathway, although subsequently less interstitial invasion has been reported [24,25]. This highlights the importance of characterising the interactions with spiral arteries since, even if there is sufficient invasion in some cases, then there could be a primary deficiency in how trophoblast are able to prime or interact with and remodel the vessels.

## 4 Problems in studying spiral artery remodelling in human pregnancies

There are a number of difficulties in studying the mechanism of human spiral artery remodelling. The process occurs over a number of weeks yet, under normal circumstances, material can only be accessed at term or following first or early second trimester terminations of pregnancy. As interventional mechanistic studies in humans are clearly impossible researchers have turned to animal models. Although the rodent model has proved useful there are limitations due to significant differences in both the depth of trophoblast invasion and the extent of spiral artery remodelling observed compared to human pregnancy. Studies of normal and ectopic pregnancies have provided valuable insights into trophoblast dependent and independent changes in spiral artery structure [14,26] but still provide little or no molecular clue as to the regulation of this important process.

Placental cells from term pregnancies and vessels from uterine biopsies taken during caesarean section or hysterectomy are available, however, their use to model events in the first few weeks of pregnancies is problematic. Cells derived from tissue at this stage will have been exposed to different stimuli and stresses and so may respond very differently to cells from the first trimester. Even within the first trimester, gestational related differences in cellular behaviour have been reported. For example trophoblasts isolated from weeks 8 to 10 are intrinsically more invasive than those isolated from 10 to 12 or 12 to 14 weeks gestation [27]. Interpretation of data from the first trimester is further complicated as a proportion of these pregnancies would have developed clinical complications had the pregnancy gone to term. A recent advance in the screening of early pregnancies by uterine artery Doppler ultrasound is starting to address this [28]. First trimester placental cells can now be isolated from pregnancies characterised according to their risk of developing pre-eclampsia. Using such an approach differences in trophoblast behaviour have been reported [29,30]. Screening of this nature will provide an extra dimension to the data obtained from first trimester material.

Studies using primary cells isolated from their in vivo environment or cell lines derived from them have been invaluable in elucidating functional aspects of trophoblast invasion and their interaction with vascular cells that would have been impossible to determine immunohistochemically. However, isolation and subsequent monolayer culture destroys three dimensional interactions with the matrix and neighbouring cells which may alter cell behaviour. For example, the trophoblast phenotype when it interacts with cells of the spiral artery will be the end result of complicated signalling events as invasion occurs; this will be difficult to recreate in cell culture studies. In recent years a number of groups, including our own, have attempted to overcome these problems by developing in vitro culture models using different matrices and formats including spheroidal cultures [31,32], complex co-cultures of different cell types and 3D explant models [33,34] which, in combination with the immunohistochemical approach, should prove particularly powerful [35]. In most of the published models trophoblast and vessels will be obtained from different patients although some attempts have been made to address this with combined chorionic villous and decidual explant cultures from the same patient [34]. In the following sections we will describe the results obtained using these models and, where appropriate, discuss any difficulties in the interpretation of the results and extrapolating to the in vivo situation.

## 5 Possible mechanisms of spiral artery remodelling

Although the details have yet to be established, several mechanisms may be involved in the changes that occur to the vascular cells and their immediate environment. These include extracellular matrix restructuring, vascular cell de-differentiation, migration, changes in cellular adhesion and sensitivity to death-inducing stimuli. These events are not mutually exclusive and may be interdependent, although one mechanism may be more important for the removal of VSMC than EC or vice versa. Some of these processes may be, in part or wholly, mediated through the actions of the invading trophoblasts and the role played by endovascular trophoblasts compared to interstitial trophoblasts may be different. It is also clear that other cells, particularly those of the maternal immune system, may play an important part in the process. Remodelling could be further modulated by the changes in blood flow and pressure that occur during the early stages of pregnancy. It is the aim of the remainder of this review to present our current knowledge of the mechanisms involved in spiral artery remodelling using data obtained from a combination of immunohistochemical studies and in vitro models. Where appropriate we will draw on the knowledge gained from vascular remodelling events that take place in other vascular beds in response to a number of physiological and pathophysiological scenarios and so highlight possible future directions.

## 6 Apoptosis and spiral artery remodelling

Apoptosis plays a role in both normal vessel regression and pathological vascular remodelling such as that seen in atherosclerosis and thrombosis [36]. Apoptosis is asynchronous in nature and, unlike necrosis, does not result in membrane disruption nor does it elicit an inflammatory response, thereby preventing local tissue damage. Apoptotic cells can be rapidly removed by neighbouring cells and professional phagocytes such as macrophages.

The fate of individual EC and VSMC within a vessel lumen is dependent on the balance between pro- and anti-apoptotic stimuli. In a quiescent vessel cell death is matched by proliferation. Cell death in excess of proliferation can lead to vascular wall instability as observed in pathological conditions such as aortic aneurism. In the spiral artery, vascular cell loss is not achieved at the expense of vessel integrity suggesting the mechanisms involved are tightly regulated. The asynchronous nature of apoptosis would fit with the gradual remodelling of the spiral arteries seen in vivo.

A number of factors are known to influence the survival of EC including growth factors, cytokines and cell adhesion. We propose that invading trophoblasts alter the balance in the vessel towards cell death, either directly via the paracrine release of apoptotic factors, or indirectly by stimulating loss of cellular adhesion (a type of apoptosis known as anoikis). As the cells of the vessel are in a steady state, loss of the endothelium together with the presence of the trophoblast could then induce VSMC death. Many of the factors that influence both endothelial and VSMC survival are present at the fetal–maternal interface and may, therefore, play a role in the remodelling of the spiral artery.

Three members of the tumour necrosis factor (TNF) family of cytokines have been implicated in the regulation of vascular cell apoptosis: tumour necrosis factor-α (TNFα), TNF-related apoptosis inducing ligand (TRAIL) and Fas ligand (FasL). All of these factors are expressed/produced by trophoblasts [37–41]. TNFα binds to and activates two distinct receptors TNF-receptor 1 (TNF-R1) and TNF-R2; both are expressed on EC and VSMC [42]. Activation of TNF-R1 leads to recruitment of the intracellular adapter molecule TNF-receptor-associated death domain (TRADD) together with a number of proteins including TRAF2 (TNF-receptor-associated factor 2). This transiently activates the JNK pathway and promotes cell survival. However, if TRAF2 recruits the Fas activating death domain (FADD), pro-caspase 8 undergoes cleavage and apoptosis is induced. The overall balance between pro- and anti-apoptotic factors present in the local environment determines which pathway is activated.

It was once thought that only transformed cells were sensitive to the effects of TRAIL; however, it is now clear that many cells including those of the cardiovascular system express the receptors on their cell surface and respond to TRAIL. TRAIL-receptor 1 (TRAIL-R1; death receptor 4 (DR4)) and TRAIL-R2 (DR5) initiate apoptosis and are expressed on EC and VSMC [41,43]. There are also two decoy receptors (DcR1 and DcR2) which are expressed by EC from different vascular beds [43–45]. Binding of TRAIL to the decoy receptors does not induce apoptosis but may compete for TRAIL binding with TRAIL-R1 and -R2 [46]. TRAIL is synthesised by first trimester cytotrophoblasts and extravillous-like trophoblast cell lines [41], and both TRAIL-R1 and R2 are expressed on VSMC and to a lesser extent on EC of first trimester spiral arteries [41].

FasL binds to and activates the cell surface receptor Fas (CD95). FasL has been detected at the maternal–fetal interface [47] where it is expressed by villous, extravillous and syncytiotrophoblast, and was proposed to contribute to the immune privilege observed in this utero–placental environment by inducing apoptosis of activated Fas-expressing maternal lymphocytes [48,49]. Fas expression has been shown in both EC and VSMC of term spiral arteries [50], however, a comprehensive investigation of its expression with gestational age remains to be carried out.

Direct co-culture techniques have been used to determine whether trophoblasts employ similar apoptotic mechanisms to facilitate spiral artery remodelling. Both primary first trimester extravillous trophoblasts and extravillous-derived cell lines induced caspase-dependent apoptosis of both EC and VSMC. Transwell co-culture experiments and incubation with trophoblast-conditioned medium indicated that this effect was, at least in part, mediated by soluble factors. FasL can be shed from the surface of cells following the action of MMPs [51–53] but whether sFasL inhibits or stimulates apoptosis is an area of some debate [51,54]. We observed that a blocking antibody to FasL/sFasL inhibited VSMC and EC death following culture with either trophoblast cells or trophoblast conditioned media, indicating a role for sFasL in this process [50,55].

In similar experiments TRAIL activation of both TRAIL-R1 and -R2 were shown to be involved in trophoblast-induced VSMC apoptosis [41]. As with FasL, TRAIL can either be a membrane associated or a soluble protein. The soluble form of TRAIL is released following proteolytic cleavage of the carboxy-terminus by cysteine proteases. However, in our studies although we could detect cell associated TRAIL in trophoblasts, we were unable to detect soluble TRAIL activity in trophoblast conditioned medium [56] or protein by specific ELISA [41]. A similar role for TRAIL in VSMC apoptosis induced by lymphocytes has been reported [57]; however, there is some controversy as in contrast to this Kavurma et al. reported increased VSMC proliferation in response to TRAIL rather than apoptosis [58].

The most common EC used in vitro studies are derived from human umbilical vein. It is now known that EC from different vascular beds can behave differently. The most appropriate cells for these studies would, therefore, be first trimester spiral artery endothelial cells, however, it has not yet possible to isolate these cells in sufficient numbers. Cells obtained from first trimester decidua can be isolated but they are a mixture of spiral artery endothelial cells and cells from other decidual vessels. In the studies detailed above both human umbilical vein endothelial cell lines and first trimester decidual endothelial cells were used and the results compared. No significant differences in the responses were observed [50].

In order to overcome some of the limitations in the use of in vitro models study the complex 3D environment we have employed the use of a more physiological model. Spiral arteries were dissected from the non-placental bed biopsies obtained at term from women undergoing caesarean section. These vessels were chosen as they would have undergone normal decidual remodelling but would not have been exposed to trophoblast, although clearly being vessels from term placenta will mean there are some differences to the vessels in the first trimester. Short segments of artery were perfused either with first trimester extravillous trophoblasts or trophoblast-conditioned medium for up to 72 h; the vessels were sectioned and stained for endothelial, VSMC or apoptotic markers. Perfusion of spiral arteries with trophoblasts and trophoblast conditioned medium lead to EC and VSMC loss by apoptosis [50,55]. Cell death was inhibited by caspase inhibitors and blocking antisera to FasL and TRAIL-R1 substantiating the effects observed in co-culture studies [41,50,55]. Subsequently Red-Horse et al. have demonstrated similar results using an in vivo model. In this study first trimester chorionic villi were transplanted to the mammary fat pads of *SCID* mice. After three weeks, EC and VSMC apoptosis were detected in arterioles associated with trophoblast invasion [59].

Within the body there are efficient mechanisms for the removal of cells undergoing apoptosis. Phagocytosis is one such mechanism known to be involved in the remodelling of other tissues. Dead and dying cells can be removed within 1–2 h of the induction of apoptosis [60–62] without apparent tissue damage. Apoptotic cells can be removed either by their neighbours or by professional phagocytes and, because cells are removed before the plasma membrane is ruptured, the release of potentially toxic or immunogenic intracellular contents is prevented. There are a number of signals that an apoptotic cell utilises to signal to phagocytes, including the phospholipid phosphatidylserine. The exposure of phosphatidylserine on the outside of a cell is a very early feature of apoptosis and precedes the nuclear events [63]. It is, therefore, apparent that cell clearance from tissues may occur before the target cells have a chance to display other characteristic markers of apoptosis [64]. In addition to professional phagocytes such as macrophages which are present in the decidua, trophoblasts are also phagocytic [65,66] and could play a significant role in removing dead and dying cells from the vessel. Other cells within the vessel including both VSMC and EC possess similar activity. The proficient clearance of apoptotic vascular cells may explain why it has proven difficult to detect apoptosis within rapidly remodelling tissues using an immunohistochemical approach [62,67]. However, recent studies of sections of first trimester decidua basalis have demonstrated apoptotic markers in both spiral artery VSMC and EC during the remodelling process [13]. As our knowledge of the pathways and sequence of events improves through in vitro studies there will undoubtedly be refinements that can be made to the snapshot immunohistochemical approach both in the timing of obtaining tissue samples along with the detection of panels of different markers. It is only when we have integrated information from both the in vitro and the in vivo situation that we can get a convincing picture of these important events; this is starting to be the case for apoptotic events in the spiral artery vascular cells.

## 7 Extracellular matrix and vascular remodelling

The structural and functional integrity of the vessel is maintained by the extracellular matrix (ECM). This is a complex structure of variable composition synthesised and maintained in a state of dynamic equilibrium by the tightly regulated activity of proteolytic enzymes. The wall of the unmodified spiral artery is formed of three distinct layers with the cells of the vessel lying on, or embedded in the matrix. The intima consists of a single layer of endothelial cells that lies on top of a basement membrane made predominantly of collagen type-IV and laminins. The elastic lamina forms a tightly woven layer of elastin and collagen type-IV fibres together with fibronectin that surrounds the intima and separates the EC and the VSMC providing strength, resilience, support and passive recoil to the vessel wall. Small pores or fenestrae within the matrix enable EC/VSMC to form direct cell to cell contact via myo–endothelial junctions. In the decidual segments of the uterine spiral arteries, the internal elastic lamina is reduced or is absent. A second prominent layer of elastic fibres surrounds the medial VSMC forming the external elastic lamina. The adventitial layer surround this and consists predominantly of collagen fibres and fibroblasts [68,69]. Both the external elastic lamina and the adventitia will act to impede the invading interstitial trophoblasts while the basement membrane and the internal elastic lamina will form a barrier to endovascular invasion and remodelling [70].

Changes in the structure of the spiral artery start before the arrival of trophoblasts [14,26] and correlate with the presence of maternal leukocytes [13]. The subsequent arrival of interstitial trophoblast is associated with changes in the myometrial segment of the spiral arteries including dilation of the lumen, intimal oedema, disruption of the elastic lamina and widening of the intercellular spaces of the media [5,71]. Further disorganisation of the intima and media takes place following the arrival of the endovascular trophoblasts which invade the sub-endothelial space disrupting EC–EC interaction and degrading elastin [22,72]. The proteolytic enzymes synthesised and released by both the vascular cells and invading trophoblasts appear central to the remodelling of the matrix. Prominent in this regard are the matrix metalloproteinases (MMPs), a family of zinc-dependent endopeptidases which together degrade all components of the extracellular matrix. MMPs are produced by EC (MMP-1 and -9), VSMC (MMP-2, -9, and the elastase MMP-12), dNK, macrophages [13] and invasive extravillous trophoblasts (MMP-1, -2, -9 -12 and membrane-type MMP) [73,74] (Table 1). Decidual natural killer cells also release granzyme, a serine–protease with trypsin-like activity that degrades a number of substrates including collagen IV and fibronectin [92,93] which may play an important role in the remodelling process.

Trophoblasts can influence the synthesis of MMP-12 by VSMC [56]. It remains to be determined whether this is an isolated effect or whether trophoblasts can affect the synthesis and release of other matrix degrading enzymes by VSMC or other cells within the environment of the spiral artery. The action of MMPs on ECM will not only affect the structure of the vessel wall but also cause the release of a number of biologically active molecules. For example, the activity of MMP-2 (expressed by both VSMC and extravillous trophoblast) can release transforming growth factor-β (TGF-β) bound to the ECM [94], while VEGF is released following the action of MMP-9. TGF-β regulates trophoblast invasion and motility [95] while VEGF is a known EC survival factor and may act to counter the apoptotic effects of the trophoblast, thereby controlling the extent of remodelling. Collagen XVIII is a minor component of the spiral artery basement membrane produced by the EC and VSMC [96]. Cleavage of collagen XVIII by MMP-2 or -9 releases endostatin, which inhibits EC proliferation [97] and stimulates apoptosis [98,99]. Both MMP-2 and -9 are expressed by the invading trophoblast [100]. Similarly the breakdown of elastin releases elastin-derived peptides which can induce the de-differentiation of VSMC and stimulate leukocyte chemotaxis, however, the role these molecules have in remodelling of spiral arteries has yet to be established [70]. In addition to a possible role for MMPs, studies in mice suggest that cysteine cathepsin 8, expressed by trophoblasts, is involved in localised spiral artery remodelling by mediating VSMC de-differentiation [101]. Whether this is the case in humans remains to be determined.

The survival of both EC and VSMC is dependent on their interaction with the ECM and the generation of specific intracellular signals. Loss of these matrix-derived signals through the direct breakdown of the ECM, or the release of inhibitory peptides following the breakdown of components of the ECM, can result in the induction of a specific form of apoptosis known as anoikis. However, apoptosis is not the only potential outcome of ECM degradation as it can also regulate cellular migration, and indeed elastase activity is associated with VSMC migration [102]. Whether the effects of matrix degradation result in migration or cell loss will no doubt depend on the extent and duration of the process. Factors affecting this have yet to be addressed.

In normal pregnancies excessive MMP activity is believed to be regulated by tissue inhibitors of matrix metalloproteinases (TIMPs) [74] produced by decidual stromal cells, EC, VSMC and trophoblasts. Although differences in both MMPs and TIMPs have been observed in cytotrophoblasts [73,103], endothelial cells [104] and maternal serum [105,106] of pregnancies complicated with pre-eclampsia direct extrapolation back to events occurring at the fetal/maternal interface in the first trimester may not be appropriate.

## 8 Fibrinoid deposition

A characteristic of the ‘physiologic change’ is the secretion of fibrinoid material composed of fibronectin, collagen type IV and laminin by extravillous trophoblasts [7,107]. Although this resembles the basement membrane, secretion is not polarised and as a result trophoblasts become embedded within the matrix. It is likely that functionally, fibrinoid deposits serve to retain the integrity of the newly remodelled vessel and may also form a basement membrane upon which re-endothelialisation can occur. Necrotic cell death is seen in the wall of modified vessels from normal pregnancies [14] and may be associated with trophoblast senescence towards the end of pregnancy [108]. The incidence of fibrinoid necrosis is increased in pathological circumstances such as pre-eclampsia where it is associated with the sub-endothelial connective tissue and muscle layers and is related to subsequent development of acute atherosis [109].

## 9 Can VSMC phenotype affect vessel remodelling?

VSMC behaviour and phenotype in any vessel will be dictated by their extent of differentiation. Unlike other muscle cells, VSMC are not terminally differentiated and can switch between ‘functional’ (contractile) and ‘synthetic’ (proliferative) phenotypes accompanied by changes in the expression of multiple genes [110]. In a healthy adult artery the majority of the VSMC exhibit a contractile phenotype expressing specific contractile proteins such as smooth muscle α-actin, smooth muscle myosin heavy chain, calponin, and smooth muscle 22α (SM22α). These cells do not generally proliferate, migrate, or secrete significant amounts of extracellular matrix [111]. However, in response to changes in extracellular cues they can adopt a more synthetic phenotype where the synthesis of components of the extracellular matrix as well as MMPs can alter. The synthetic phenotype is often, but not always, coupled with increased migration and proliferation [112] but is always associated with a loss or reduction in the expression of SMC-specific contractile proteins. In regard to spiral artery remodelling a change in VSMC phenotype could result in an increase in the sensitivity to apoptotic stimuli [113,114] or increased migration away from the vessel; indeed de-differentiation of VSMC is known to increase their migratory potential [79]. Loss of a contractile phenotype is associated with altered vessel wall structure, including loss of the layered organisation of VSMC, migration away from the lumen and loss of differentiation markers in other examples of vascular remodelling such as atherosclerosis [79,115].

The differentiated state of a VSMC is dependent on a number of external factors including neighbouring cells and the ECM [116,117]. EC promote VSMC differentiation, stimulating the expression of contractile proteins [118], and at the same time inhibit both VSMC matrix deposition and proliferation [119], in part mediated by the signalling molecule nitric oxide (NO). Trophoblasts can release key inflammatory cytokines including TNFα which together with IL-1β could stimulate the expression of the inducible form of nitric oxide synthase (iNOS) in VSMC, which could result in increased VSMC apoptosis [120].

## 10 Haemodynamic influences on vessel structure and remodelling

The interactions between the EC and VSMC are influenced by both mechanical factors such as shear stress and pressure and paracrine and autocrine signals. These signals are derived either from within the vessel wall itself or the surrounding tissue and affect fundamental cellular properties such as the state of differentiation and cell survival. Disturbance of these interactions, either as a result of the haemodynamic changes that may accompany pregnancy, or the presence of invading trophoblasts could have a role to play in the remodelling that takes place. Although these issues are only just beginning to be addressed in regard to spiral artery remodelling there is already a substantial literature concerning the effect of these factors on vessels in other vascular beds.

The haemodynamic changes that take place in spiral arteries undergoing transformation are considerable. Formation of the endovascular trophoblast plug in the first few weeks of gestation [121] will dramatically reduce blood flow in these vessels and at the same time increase the transmural pressure. As the pregnancy progresses, the pressure within the plugged vessel will fall as the VSMC in the myometrial segments lose their structured organisation, a process that correlates with interstitial trophoblast density [5]. With the loss of integrity of the plug the flow of blood to the intervillous space increases as does the shear stress experienced by the vessel wall.

How such haemodynamic changes might influence spiral artery remodelling has not been established, however, the effect of retrograde blood flow on endovascular trophoblast migration and invasion has been discussed [122]. It is highly likely that the response of vascular cells to invading trophoblasts is modulated by the haemodynamic stresses placed upon them. As remodelling progresses throughout gestation these stresses change and so, therefore, will the remodelling response. Evidence from other vascular systems illustrate the profound effect that mechanical forces can have on the structure of a vessel by significantly altering EC and VSMC differentiation, growth [123] and response to external mediators. Physiological levels of shear stress inhibit endothelial apoptosis induced by a number of stimuli including TNFα and oxygen radicals [124,125]. It is, therefore, possible that the loss of laminar flow following trophoblast plugging of the spiral artery could increase EC susceptibility to trophoblast-mediated apoptotic stimuli. Haemodynamic forces also influence interactions between cells of the vessel wall: for instance EC modulate VSMC phenotype which affects matrix deposition [126] whereas VSMC can influence the EC response to shear stress [127,128]. Although the VSMC may also be influenced by shear stress, they are exposed more to mechanical stretch. In many vessels including the unmodified spiral artery this will be experienced in pulses. Unlike EC, the effect this stimulus has on VSMC proliferation and apoptosis is less clear-cut and may depend on the frequency of the pulse and/or the degree of VSMC differentiation [129,130].

## 11 Role of natural killer cells in vessel remodelling

Decidualisation is associated with a large infiltration of maternal natural killer cells with a specific phenotype that distinguishes them from peripheral blood NK cells. These cells are present at a time when spiral artery remodelling is occurring and then decrease in number from mid-gestation [131]. These decidual NK (dNK) cells form the major component of immune cells within the decidua basalis (approximately 70%) with macrophages forming the next most abundant immune cell type (20–30%) followed by a low number of T cells. Immunohistochemical studies in humans suggest that some of these dNK are localised near the decidual regions of maternal spiral arteries [132]. However, NK cells are absent in the inner myometrium hence remodelling in this area is unlikely to be affected by these cells [133].

In addition to roles in interacting with extravillous trophoblast and in cytokine secretion (reviewed by Tabiasco et al. [134]) studies in mice have indicated involvement of dNK cells in the regulation of spiral artery remodelling. Ashkar et al. [135] demonstrated that mice depleted of dNK cells show inadequate spiral artery remodelling a process regulated, at least in part, by production of interferon-γ (IFNγ). This has yet to be elucidated in humans but, if it is the case, then at least two possibilities exist to explain how dNK/IFNγ could affect vascular cells. There could be a direct effect from a dNK-derived factor on the vascular cells (perhaps IFNγ, but not necessarily the only factor) or, as there is considerable cross-talk between extravillous trophoblasts and dNK cells, dNK may regulate the trophoblast-induced remodelling events. Studies in humans have shown that dNK cells can produce other cytokines in addition to IFNγ including TNF-α, leukaemia inhibitory factor (LIF), CSF-1 and IL-8 [136,137]. IFNγ can cause both proliferation and apoptosis of VSMC [138]; events of importance to remodelling in other vascular beds and to the development of arteriosclerotic lesions. IFNγ can act to prime VSMC for death-receptor induced apoptosis [139], which could be relevant to spiral artery remodelling given the local environment of pro-apoptotic factors produced by trophoblast and, in the case of TNFα, additionally by dNK cells. In addition, IFNγ can increase TRAIL production by trophoblast [140]. Decidual NK cells can produce many MMPs which could degrade the matrix proteins as discussed above [83], thus affecting vessel stability, as well as IFNγ modulating ECM protein production directly [141]. Recent immunohistochemical studies relating the localisation of immune cells (dNK and macrophages) with the stage of spiral artery remodelling suggest that the immune cells may have a role in earlier stages causing the disorganisation of vascular cells and separation of smooth muscle layers prior to the arrival of the interstitial and endovascular trophoblast [13].

Human dNK cells can also produce a variety of angiogenic factors, including VEGF, placental growth factor (PLGF) and angiopoietin-2 (Ang-2) [137]. There may well be parallels between the regulation of angiogenesis, defined as the remodelling of an existing vascular network, with the events seen in spiral artery remodelling, although the direct involvement of these factors has yet to be determined.

## 12 Co-ordination of cellular and molecular mechanisms involved in spiral artery remodelling

**Fig. 1:**
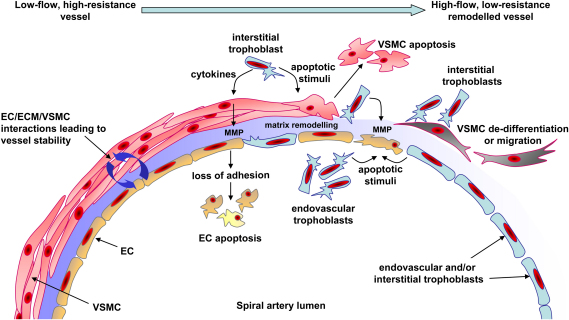
Diagram of possible mechanisms involved in trophoblast-dependent spiral artery remodelling. Prior to remodelling low-flow, high-resistance spiral arteries are maintained in a stable state by interactions and signalling between endothelial cells (EC), the extracellular matrix (ECM) and vascular smooth muscle cells (VSMC). The decidualisation process causes EC vacuolation and VSMC swelling (not shown); however, the major changes to vessel structure occur after extravillous trophoblast reach the vessel. Trophoblasts invading interstitially would interact with VSMC first while endovascular trophoblasts would initially encounter EC in the vessel lumen. Trophoblasts can produce pro-apoptotic factors which can induce vascular cell apoptosis. Apoptotic cells may then be rapidly removed by professional phagocytes such as macrophages or other cells, such as trophoblasts, which also possess phagocytic activity. Proteolytic enzymes produced by trophoblasts and vascular cells can influence the composition of the ECM proteins important in maintaining vessel integrity. In particular matrix metalloproteinases (MMP) can be produced by trophoblasts and their production by VSMC can be regulated by trophoblasts. Loss of adhesive interactions between vascular cells and the remodelled ECM could then lead to vascular cell apoptosis. Changes in the ECM or signals from trophoblasts may influence the state of differentiation of VSMC by promoting a switch from a contractile to a more synthetic, proliferative phenotype, which may also be accompanied by increased migratory activity and sensitivity to pro-apoptotic factors. Haemodynamic factors and the presence of decidual natural killer cells will also play a role in the regulation of remodelling (not shown). The high-flow, low-resistance remodelled vessel will consist of trophoblasts embedded in a fibrinoid material as a replacement for the VSMC. The endothelium is temporarily replaced with a trophoblast layer, although it is restored later in pregnancy.

So far the various mechanisms that might be involved in spiral artery remodelling have primarily been considered in isolation of each other. However, it is clear that a number of these mechanisms are interdependent (summarised in Fig. 1). In many vascular beds the sensitivity of VSMC to apoptotic stimuli is influenced by factors that alter their state of differentiation, which is in turn influenced by the composition of the ECM and signals derived from the EC. The way EC behave will be determined by signals from the VSMC and the ECM on one side and the physico-mechanical stresses of shear and stretch on the other. All these factors and more will influence how these cells within the vessel respond not only to the invading trophoblasts but also the infiltrating maternal immune cells.

It is also apparent that differentiation will affect the synthesis and release of proteolytic enzymes. This will change the composition of the ECM and this in turn will alter the signals received by the vascular cells which will further influence their differentiated state. Loss of the endothelium as a result of trophoblast invasion will also have a direct effect on VSMC differentiation and survival through the loss of important survival and differentiation signals, perhaps making them more sensitive to the apoptotic signals released by trophoblasts. In addition to vascular cell apoptosis, migration of VSMC away from the vessel may provide another mechanism for the reduction of VSMC associated with the remodelled spiral artery, although this hypothesis requires further investigation.

In recent years the processes involved in the crucial pregnancy adaptation of spiral artery remodelling have begun to be elucidated. Many groups have developed novel approaches to model these events in vitro which, in combination with previous histological studies, are giving insights into the regulation of spiral artery remodelling. Continued research in the placental field combined with lessons learnt from studies in other vascular beds will allow the exact role of trophoblast-dependent and -independent changes to be determined. It is only when we have a clearer picture of the events occurring in a normal pregnancy that we can begin to determine which of these are compromised in pregnancies complicated by disorders such as pre-eclampsia.

## Acknowledgements

The authors would like to acknowledge the financial support of The Wellcome Trust (Project grant 069939) and The British Heart Foundation (Project grants 2001045 and 05126).

## Figures and Tables

**Table 1:** Expression of MMPs and TIMPs by trophoblasts, immune and vascular cells.

MMP	Enzyme	Substrate	Cell type
I	Collagenase-1	Collagen type I, II, III, VII, VIII, X, MMP-2 and -9	EC reviewed [75]
			iEVT^a^[76,77]

2	Gelatinase A	Collagen type I, II, III, IV, V, VII, X, XI, elastin, fibronectin, MMP-9 and -13	VSMC (synthetic phenotype)^b^[78] reviewed [79,80]
			EC reviewed [75,81]
			iEVT^a^[82]
			dNK^a^[83]
			SGHPL-4 EVT cell line [84]

3	Stromolysin-1	Collagen type III, III, IV, IX, X, XI, elastin, fibronectin, laminin and MMP-7, -8 and -13	iEVT^a^[77,85]
			SGHPL-4 EVT cell line^b^[84,85]

7	Matrilysin-1	Collagen type IV, X, elastin, fibronectin, laminin and MMP-1, -2, and -9	dNK [13]
			Macrophage^a^[82]
			vEVT^a^[82]

9	Gelatinase B	Collagen type IV, V, VII, X, XIV, elastin, fibronectin	VSMC (synthetic phenotype)^b^[86] reviewed [79,80]
			EC reviewed [75,81]
			Cytotrophoblast^b^[87–89]
			vEVT^a^[82]
			SGHPL-4 EVT cell line [84]
			dNK^a^[13,83]
			Macrophage [82]

10	Stromolysin-2	Collagen type III, IV, V elastin, fibronectin, laminin and MMP-1 and -8	VSMC reviewed [79,80]
			EC reviewed [75,81]

12	Metalloelastase	Collagen type IV, elastin, fibronectin and laminin	VSMC reviewed [79,80]
			EVT^b^[90]
			BeWo cell line [56]

13	Collagenase-3	Collagen type I, II, III, IV	SGHPL-4 EVT cell line [84]

14	MT1-MMP	Collagen type I, II, III, elastin, fibronectin, laminin, MMP-2 and -13	EVT^a^[91]
			SGHPL-4 EVT cell line [84]
			Cytotrophoblast^b^[87]
			VSMC reviewed [79,80]
			EC reviewed [81]

24	MT5-MMP	Fibrin	SGHPL-4 EVT cell line [84]

TIMP	Inhibition	Cell type
I	All MMPs	VSMCa,b[142]
		EVT^a^[76,83]
		Cytotrophoblast^b^[87]
		iEVT [83]
		dNK [83]

2	MMP-2 and MT1-MMP	EC reviewed [81]
		VSMCa,b[142]
		iEVT^a^[77]
		Cytotrophoblast^b^[87]
		dNK^a^[83]

3	MT1-MMP	dNK^a^[83]
		Cytotrophoblast^b^[87]

iEVT: interstitial extravillous trophoblast, and vEVT: endovascular extravillous trophoblast.

a In vivo studies.

b In vitro studies.
